# Medical Legal Points

**Published:** 1911-10-21

**Authors:** 


### MEDICAL LEGAL POINTS.

### I.—Death from a Medico-Legal Aspect.

Death, even when it is the consequence of disease, is often an unexpected event. But if an individual expires under his own roof, surrounded by friends and relatives, we are disposed to consider it as an ordinary dispensation of Providence, and one to which all of us are sooner or later doomed. The features of the case differ materially when a person is found dead on the highway, on the banks of a river, or in a lonely place. Indeed, if he be discovered to have paid the last debt of mortality, either in a sudden manner or at a distance from his home, the laws of civilised society demand an investigation of the cause, and over this investigation the officer called a coroner is appointed to preside.

### The Question of Death.

The opinion of the physician as to the reality of a death may be sought under the following circumstances: (1) Where the person has directed by will that before being finally "screwed down" he shall be certified by a competent authority to be dead; (2) where, owing to certain exceptional occurrences, such as post-mortem hæmorrhages, the turning of a body in the coffin and other effects resulting from the development of the gases of putrefaction, or to the slowness with which from accidental causes putrefaction may develop itself, or to the unaltered and life-like condition of the face and countenance not infrequent after certain forms of death, or to the retardation of post-mortem cooling, a medical opinion confirmatory of the reality of the death is sought by the relatives; (3) where, in cases of succession to titles or large property, the relatives require a certificate of the reality of the death; (4) and lastly, cases arise where, a dead body being found, the opinion of the medical jurist as to the interval that has elapsed since death.

The medical jurist, in determining the reality of death, must convince himself not only of the entire cessation, but of the continuous cessation, of the action of the heart; for the heart may seem to cease for a time, yet after a brief interval recommence action. To decide the absolute absence of all cardiac sounds, auscultation and palpation in a perfectly quiet room are necessary. And this examination must include not merely the regions of the heart proper, but of the chest generally, remembering that both the position and the sounds of the heart may be abnormal. Assuming that this absence of cardiac sounds is continuous, and not merely accidental, it should be determined by careful auscultation for two or three hours, at intervals of fifteen minutes. There can be no doubt that recovery has taken place after the heart has, at any rate apparently, ceased beating for a quarter of an hour. In the case of children, efficient means have succeeded in restoring vitality where for fifteen or thirty minutes after birth no pulsations could be detected.

Although the fact of death is to be determined by no single sign but by a combination of several tests, the entire and the continuous cessation of the heart's action undoubtedly ranks first in importance. Breathing being a vital act, any long suspension of this function must prove fatal. The time that people can "hold their breath" varies greatly, but three minutes and a half may be taken as a maximum. This, however, refers only to the absolute and voluntary cessation of breathing. To commit suicide by holding one's breath is a practical impossibility. It is certain that life may be prolonged considerably beyond three and a half minutes when the stoppage is involuntary, or when the slightest attempt at respiration, however slow and shallow, is made.

### Somatic and Molecular Death.

It is very necessary for the medical jurist thoroughly to appreciate the importance of the distinction between systematic or somatic death and death of the tissues or molecular death. In profound syncope, for example, there is every external appearance of death; but if the heart resumes its function, the patient recovers. Hence the necessity of careful study of the phenomena which succeed death in order to be able to distinguish between real and apparent death. In awarding the Manni prize, founded for the discovery of a certain sign of death, the French commissioners very properly dwelt upon the state of the heart as furnishing the most unequivocal proof of death before the occurrence of cadaveric rigidity and putrefaction. Bouchut, to whom the prize was awarded in 1846, found, in an extensive series of researches, experimentally confirmed by the commissioners themselves, that in all cases of apparent death, whether arising from asphyxia or syncope, there is one common character by which they may be distinguished from real death, and that is a continuance of the pulsations of the heart. He established the fact that in the most perfect state of syncope, attended with entire loss of motion and sensation, as well as cooling of the body, the contractions of the heart were not really at any time suspended, but simply reduced in force and frequency. In syncope from hæmorrhage carried to the fullest extent, and in cases in which respiration was either imperceptible or carried on at long intervals, the body at the same time having the aspect of a corpse, he was enabled by auscultation to detect the pulsations of the heart, and thus to distinguish apparent from real death. In children born in a state of apparent death and in cases of asphyxia from any cause, in narcotic poisoning, in hysterical and epileptic coma, and in all diseases which have been stated to resemble apparent death, the living has been easily distinguished from the dead body by the continuance of the heart's action. This was feeble and took place at intervals, but it was always sufficiently marked to enable a professional man to distinguish a living from a dead body.

A number of tests have been devised in order to determine whether the circulation has or has not ceased, such as examining the hand of the supposed dead person by transmitted light. During life the margins, the tips, and the web of the fingers appear red and translucent; after death the fingers appear opaque up to their edges. On tying a piece of string round a finger its distal end becomes swollen and bluish-red if the circulation continues; if it has ceased, no effect is produced.

After the occurrence of somatic death—that is, after permanent cessation of respiration and circulation—certain phenomena occur in definite order. The time occupied by many of these phenomena, although variable, is limited; and consequently, by ascertaining the stage at which they have arrived, an opportunity is afforded of approximately estimating the interval that has elapsed between death and the period of investigation. These phenomena are manifestations of the occurrence of molecular death. The rapidity of their onset is to some extent determined by conditions which exercise an influence on the amount of vital energy with which the tissues are endowed immediately before somatic death. Such conditions comprise disease, poison, violent exercise, and other exhausting influences. If the tissues are depressed in vitality at the moment of somatic death they rapidly succumb to molecular death, and the course of the succeeding phenomena is hastened. If, on the other hand, they are in full activity when somatic death takes place, they resist the advent of processes which are really the antecedents of decomposition.

During life the healthy body has a warmth (independently of the surrounding temperature) of from 97° to 100° F. After death a gradual and progressive loss of heat occurs both externally and internally, until in time the temperature of the body becomes almost the same as that of the medium by which it is surrounded. Post-mortem cooling, however, derives its special medico-legal importance from the fact that this loss of heat is progressive. Temperature thus becomes, in many cases, not merely a sign of death, but an indication of the time that a body has been dead. All observations on temperature should be thermometric. In forensic cases both the external and the internal temperature should be recorded. The external temperature, as a rule, sinks more rapidly than the internal, the skin and solids of the body generally being bad heat conductors. Again, the temperature should be taken at stated intervals, remembering that it is not the absolute temperature which is so important medico-legally as the progressive and continuous cooling of the body. In ordinary cases a body becomes cold in from fifteen to twenty hours after death. In certain cases of disease, or where the body has been freely exposed to air, draughts, etc., the cooling process may practically be complete after four to five hours; whilst in certain other exceptional diseases, or under unfavourable conditions for cooling, forty-eight hours, or even three days, may elapse before the body is cold. Of all the changes that occur in the dead body, that of cooling down to the temperature of its surroundings is the one about which we have the most knowledge. Its rate with very few exceptions, obeys laws which are comparatively well understood, and it is perhaps the most reliable change by which to calculate the time since death.

After death the body parts with its internal heat by radiation, so that the superficial temperature may rise above that which existed before death. Usually, there is no further generation of heat when life is extinct, but in certain cases, as in death from cholera or yellow fever, and some cerebro-spinal diseases, the temperature of the dead body has been observed to rise considerably above the normal temperature of life. Nor is the extinction of animal heat a sure means of determining the time of death; for the rate of cooling varies with the age, the cause of death, the treatment of the body itself, and the state of the atmosphere; so that the period of cooling may vary from two or three hours to fifteen or twenty, and may even extend to upwards of four days. In death from chronic diseases, where there has been great wasting (phthisis, cancer, œsophageal disease, etc.), in cholera, after great losses of blood, etc., the body usually cools rapidly—so rapidly, indeed, that in three, four, or five hours its external temperature may be almost identical with that of the surrounding medium. Of other causes of rapid cooling may be mentioned: The body being exposed to air and cold draughts in a more or less uncovered state. The size of the apartment where the body lies, cooling being more rapid in a large and draughty room than in a small, warm one. The bodies of children and of old persons cool more rapidly than those of ordinary adults. The bodies of lean people cool more rapidly than those of stout.

For some time after somatic death the muscles retain their irritability, and contract if stimulated. The duration of this irritability does not, as a rule, extend beyond two or three hours, and when it ceases cadaveric rigidity sets in. Before, however, rigidity becomes marked, the irritability of the muscles may be restored by the injection of defibrinated arterial blood or aerated venous blood through the vessels. The irritability lasts longer in a low temperature than a higher one. Muscles become rigid at high temperatures. So long as the muscles remain flaccid they retain their irritability, and respond to electrical stimuli in the same way that they do in life; this is due to the persistence of molecular or tissue life after the cessation of somatic life. At a variable period after death the muscles of the lower jaw begin to stiffen; this is the first indication of the onset of cadaveric rigidity, or, as it is also called, "rigor mortis." Under ordinary circumstances the skeletal muscles begin to stiffen in from four to ten hours after death. The stiffening spreads from the muscles of the jaw to those of the face, neck, and trunk, and lastly to the limbs. It is fully developed in from two to three hours, when the entire body is firm and stiff. The limbs cannot be flexed at their joints without considerable force, and the body when moved behaves as though it was devoid of articulation.

(*To be continued.*)

